# Nurses’ reflections on caring for sexual and gender minorities pre-post stigma reduction training in Uganda

**DOI:** 10.1186/s12912-023-01208-w

**Published:** 2023-02-23

**Authors:** Patience A. Muwanguzi, Racheal Nabunya, Victoria M. S. Karis, Allen Nabisere, Joan Nangendo, Andrew Mujugira

**Affiliations:** 1 Department of Nursing, School of Health Sciences, College of Health Sciences, Makerere University, Kampala, P. O. Box 7072, Uganda; 2 Clinical Epidemiology Unit, School of Medicine, College of Health Sciences, Makerere University, Kampala, P.O. BOX 7072, Uganda; 3 Infectious Diseases Institute, College of Health Sciences, Makerere University, Kampala, P. O Box 22418, Uganda

**Keywords:** Men who have sex with men, Nurses, Qualitative, Stigma reduction, Sub-Saharan Africa, Transgender people

## Abstract

**Background:**

Men who have sex with men (MSM) and transgender women (TGW) have a significant HIV burden worldwide. Data from eight countries across sub-Saharan Africa found a pooled HIV prevalence of 14% among MSM and 25% among TGW. Stigma and discrimination among healthcare providers are barriers to healthcare access by these populations. We sought to explore nurses’ attitudes before and after sensitivity training to reduce stigma in HIV prevention and care provision to MSM and transgender persons in Uganda.

**Methods:**

An explorative qualitative study comprising in-depth interviews. Nineteen nurses who underwent sensitivity training in caring for the vulnerable, priority and key populations in Uganda participated. We interviewed each participant before and after the stigma reduction training and analyzed the data with NVivo.

**Results:**

Eight (8) themes emerged from the reflections before the training, namely, ‘the definition of MSM and transgender persons’, ‘legal concerns’, ‘mental illness’, ‘attitude in health care provision’, ‘personal perceptions’, ‘self-efficacy’, ‘insufficient training preparation’, and ‘reasons for gender or sexual orientation preference’. The post-training reflections suggested a change in knowledge and attitude. Five themes emerged for MSM: ‘stigma reduction’, ‘sexual practices and sexuality’, ‘the need for tailored health approaches’, ‘MSM and the law’ and ‘corrected misconceptions’. For transmen, ‘reproductive health needs’, ‘social needs’, ‘safety needs’, ‘Gender identity recognition’ and ‘reduced stigma, discrimination, and barriers to care’. Finally, the reflections on their attitudes towards transwomen were on five topics; Gender affirming care’, ‘Healthcare provision for transwomen’, ‘Need for further training’, ‘New knowledge acquired’, and ‘Sexual violence’.

**Conclusion:**

Nurses’ attitudes and empathy for vulnerable and key populations improved following the training. Nursing training programs should consider incorporating sexual and gender minority (SGM) specific health training into their curricula to decrease negative attitudes. There is a need to identify best practices and conduct implementation research to provide culturally sensitive and affirming healthcare delivery in sub-Saharan Africa. Future studies should evaluate the effect of provider sensitivity training on sexual health and HIV outcomes for SGM. Furthermore, interventions targeting higher-level stigma, such as structural and policy levels, are critical because they influence interpersonal stigma reduction efforts and initiatives.

**Supplementary Information:**

The online version contains supplementary material available at 10.1186/s12912-023-01208-w.

## Background

Sexual and gender minorities (SGM), including men who have sex with men (MSM) and transgender people (TGP), account for disproportionally high rates of new HIV infections [1]. Gay men and other MSM have a 28 times greater risk of acquiring HIV than adult men (15–49) in the general population and this risk is 14 times higher for transgender women compared to other adults aged 15–49 years [2]. Despite the high disease burden, wide gaps exist in addressing the health needs of sexual and gender minorities. The legal systems that criminalize MSM and the central identity of TGP compound this lack of attention to their specific health needs. Additionally, there is a lack of gender-affirming legislation, including failure to offer protection against discrimination [3, 4]. Several types of stigma exist among these populations. Enacted stigma - the experience of unfair treatment by others - is common in healthcare settings in sub-Saharan Africa [5], where lack of provider knowledge about culturally sensitive care is a significant barrier to healthcare access by SGM. This situation is not any different in several parts of the world, as highlighted by studies done in several countries [6–12].

Nurses and midwives in these settings do not receive formal training in SGM healthcare and have a myriad of attitudes, values and beliefs which affect their care of MSM and TGP [13]. In Tanzania and Uganda, SGM clients reported stigma and discrimination, fear of exposure, limited MSM-safe HIV-related health services and stigmatizing attitudes from health workers [14, 15]. Other work from Zambia confirms a high prevalence of condemnatory attitudes toward MSM and TGP among varying groups of health providers [16]. In South Africa, healthcare providers described their attitudes towards MSM and TGP as having a low proficiency, training and expertise to support their health needs and vulnerabilities [14]. MSM and TGP verbalized stigmatizing experiences, guilt, and a sense of embarrassment and indignity resulting from stigma and discrimination by health workers [17]. In Kenya, healthcare workers lacked knowledge of the management of MSM sexual health needs and marked homophobia [18]. Increasing the accessibility of MSM and TG-centered and friendly health services and training health providers to be empathetic and uphold people’s right to health care decreases enacted stigma [19].

Implementing targeted stigma and discrimination mitigation interventions in health provision settings is key to meeting the health needs of vulnerable SGM [16]. Attitudinal change, increased understanding of SGM as more than their sexual behaviours, a reduction in prejudices towards SGM, and improved self-efficacy for appropriate health provision are crucial to addressing the health challenges of this high HIV-risk population. This change can is achievable through stigma reduction training and other interventions [20]. Several stigma-reduction interventions for SGM exist in Sub-Saharan Africa. These include training health workers in cultural responsiveness and involvement of SGM peer educators in Uganda [21], and addressing structural stigma through Advocacy and Other Community Tactics in Burundi, Cameroon, Côte d’Ivoire, Ghana, and Zimbabwe [22]. Other interventions include the reduction of interpersonal stigma in Ghana through participatory stigma-reduction training for all staff levels with delivery by staff and clients from the facilities who were trained as stigma-reduction facilitators [23] and a participatory theatre intervention in Eswatini and Lesotho [24]. The intervention in Uganda resulted in 90% retention rates for MSM and 81% retention for transgender women at 12 months in a clinical trial where the participants reported a conducive and welcoming environment [21]. The study in Ghana did not report any statistically significant difference-in-differences between the intervention and control facilities on the composite stigmatizing attitudes variable [25]. Finally, in addition to the positive outcomes, the multi-country study reported some undesirable outcomes of the study including loss of income and stigma among the participants [22]. There is limited information on the participants’ reflections, specifically nurses, who are usually the first contact for patients at health facilities before and after these interventions.

Therefore, this study sought to determine nurses’ perceptions of caring for SGM before and after the training to develop further interventions if the existing ones play a role in stigma reduction towards these populations. This study sought to qualitatively explore changes in perceptions of and knowledge about MSM and TGP following stigma reduction training among nurses before and after they complete the training.

## Methods

The underlying philosophy influencing the choice of research methods is pragmatism. This philosophy is appropriate because there is very little known about this topic. Therefore, this flexibility will allow the study to culminate with rhetoric about the nurses’ reflections on caring for SGM (Creswell & Poth, 2016).

### Study setting and participants

Registered and enrolled nurses from all regions of Uganda were purposefully selected to attend a week-long stigma reduction training workshop in Kampala. We included those working at primary, secondary, and tertiary healthcare settings in Uganda as well as from the public and private sectors.

### Training workshop information

We leveraged existing stigma reduction training workshops for health workers that are organized by the government or non-governmental organizations in Uganda. These are on-going sessions for health professionals in HIV care of SGM in Uganda. The workshops follow the Most At Risk Populations Initiative (MARPI) HIV training approach for sensitization about caring for vulnerable, priority and key populations (KP) [5]. This content focuses on the HIV burden among KP - MSM, TGP (especially transgender women), sex workers, people who inject drugs, and prisoners and other incarcerated people - as well as adolescent girls and young women, and the vulnerabilities that increase their HIV risk. The training content also includes modules for health workers on stigma-free care, offering non-discriminatory care to increase the uptake of HIV prevention as well as a module on the prevention of gender-based violence. The training is based on existing evidence that training health professionals results in improved comfort and confidence in working with SGM populations [26]. For example a training intervention reported significant improvement in health provider’s post-intervention scores in knowledge, attitudes/biases and perceived competency in transgender-affirming HIV care (score mean difference (MD) 8.49 (95% CI of MD: 6.12–10.86, p < 0.001, possible score range: 16–96) [27]. The basic training package lasts 5 days, however, longer training periods are available for health professionals who work specifically with key populations. For this study, we interviewed nurses who attended the 5-day training sessions between April and June 2022. The average attendance of each 5-day session was 10–15 participants and we interviewed nurses from 7 different training sessions around Uganda. For this paper, we focus on the nurses’ reflections on caring for MSM and TGP. Key population peer educators led several sessions, shared personal stigmatizing experiences in healthcare settings and held interactive question and answer sessions with trainees.

### Data collection

Data were collected using in-depth interviews. We conducted each interview per the Ugandan Ministry of Health’s COVID-19 standard operating procedures. We interviewed each participant before and after sensitization training. Initially, we contacted community-based organizations focusing on ending discrimination and violence against LGBTQI + people. These non-governmental organizations offer HIV prevention and care to KP and the Ugandan Ministry of Health regarding sensitivity or stigma reduction training for health workers in Uganda. The organizations that responded positively provided a training curriculum. We sought and received their permission to interact with the nurses attending subsequent training workshops.

On the first training day, authors PAM and RN met with the nurses to discuss their expectations and perceptions before the training. Each nurse provided informed consent before participating in qualitative interviews. Similarly, a post-training interview was conducted on the last day of the training or during the next week for nurses who wanted to process what they had learned. The interviews were conducted between April and June 2022, each lasting 45–60 min. We held the pre-training interviews privately at the training venues while the participants selected the post-training interview locations. The same nurses participated in the pre-and post-training interviews.

We used a semi-structured interview guide designed by authors PAM, RN, MVSK and AN. For the pre-training interview, we sought data on nurse definitions of MSM and TGP, attitudes and perceptions towards these communities and perceptions of offering care to both populations at health facilities. The post-training guide covered knowledge attained and any differences in attitudes and perceptions of offering care to KP at health facilities. Saturation determined data collection; there was enough information to replicate the study, and we did not attain any new information [28].

Both interview guides were pilot tested with three nurses from similar settings. Interviews were conducted in English, audio recorded with participant consent, and transcribed verbatim by VMSK.

### Data analysis

Data were analyzed using NVivo [29] and followed the ‘data analysis spiral’ procedure proposed by Creswell and Poth [30]. The five steps in data analysis spiral method include (i) managing and organizing data, (ii) reading and memoing emerging ideas, (iii) describing, and classifying codes into themes, (iv) developing and assessing interpretations and (v) representing and visualizing the data.

In our study, the first step involved organizing the data into digital files; we created a file naming system to include participants’ IDs and session numbers. Additionally, this step involved ensuring long-term and secure storage of the files on a password-protected server. Second, the team read the transcripts several times to get acquainted with each interview before breaking it into parts. We transcribed the recordings verbatim, and the team reviewed the written transcripts multiple times. While reading, statements, words, or paragraphs revealed meaningful reflections on the central phenomenon highlighted. Third, we started describing, classifying, and interpreting the gathered data, including gathering highlighted meanings, paraphrasing, and assigning themes to the meanings. PAM, AN and RN independently built detailed descriptions, applied codes, developed themes or dimensions, and provided an interpretation considering their views or perspectives in the literature.

Fourth, the team compared related themes and discussed which to cluster according to an essential meaning or experience. We looked for frequently used words or phrases, and a descriptive picture was drawn of each participant’s considerations, as reflected thematically across each transcript. Participant summary experiences were compared to build overarching themes. We abstracted beyond the codes and themes to the more significant meaning of the data. In the final phase of the spiral, we presented the data in tabular or figure form, alongside narrative quotes from the participants. Following this process, three expert participants reviewed the codes and themes to ensure they represented the actual and intended meaning.

The rigor of the data was guided by the four trustworthiness criteria [31]. Techniques such as openness, immediate transcription, and saturation established the credibility of the results [32]. Furthermore, the entire research team had prolonged engagement with the study participants and established trust and rapport. Additionally, three nurses corroborated the resulting categories and themes as part of member checking. For dependability, recordings, and transcripts established an audit trail, clearly presenting the step-by-step data collection and analysis process. For transferability, thick descriptions have been provided to allow the reader to judge whether the findings apply to their contexts. Documenting the researchers’ reflections ensured the confirmability of the study.

## Results

### Study sample characteristics

Twenty-five nurses were approached to participate and six declined. Nineteen (19) nurses participated in the interviews. The mean number of years in clinical practice was 9 (SD 5.86), with the majority still within the first decade of practice. The highest nursing qualification attained was a master’s degree. All the nurses had heard about MSM, but only 21.1% had provided health care to a person who identified as MSM, 36.8% had heard of transgender women, 10.5% had provided healthcare to a transgender woman, while none had offered treatment to a transgender man (Table 1).

**Table 1:** Participant Characteristics (N = 19)

Number of years of clinical practice (years)	Mean	SD
Mean	9	5.86
**Number of years of clinical practice (years)**	**n**	**%**
1–5	7	36.8
6–10	6	31.6
11–15	3	15.8
16–20	2	10.5
> 20	1	5.3
**Gender identity**		
Male	3	15.8
Female	15	78.9
Prefer not to say	1	5.3
**Highest nursing qualification**		
Certificate	4	21.1
Diploma	6	31.6
Bachelor’s degree	5	26.2
Master’s degree	4	21.1
**Knowledge about key populations**		
MSM	19	100
Transgender women	7	36.8
Transgender men	1	5.3
**Knowingly provided health care for key populations**		
MSM	4	21.1
Transgender women	2	10.5
Transgender men	0	0

### Nurses’ pre-training reflections

**Fig. 1:**
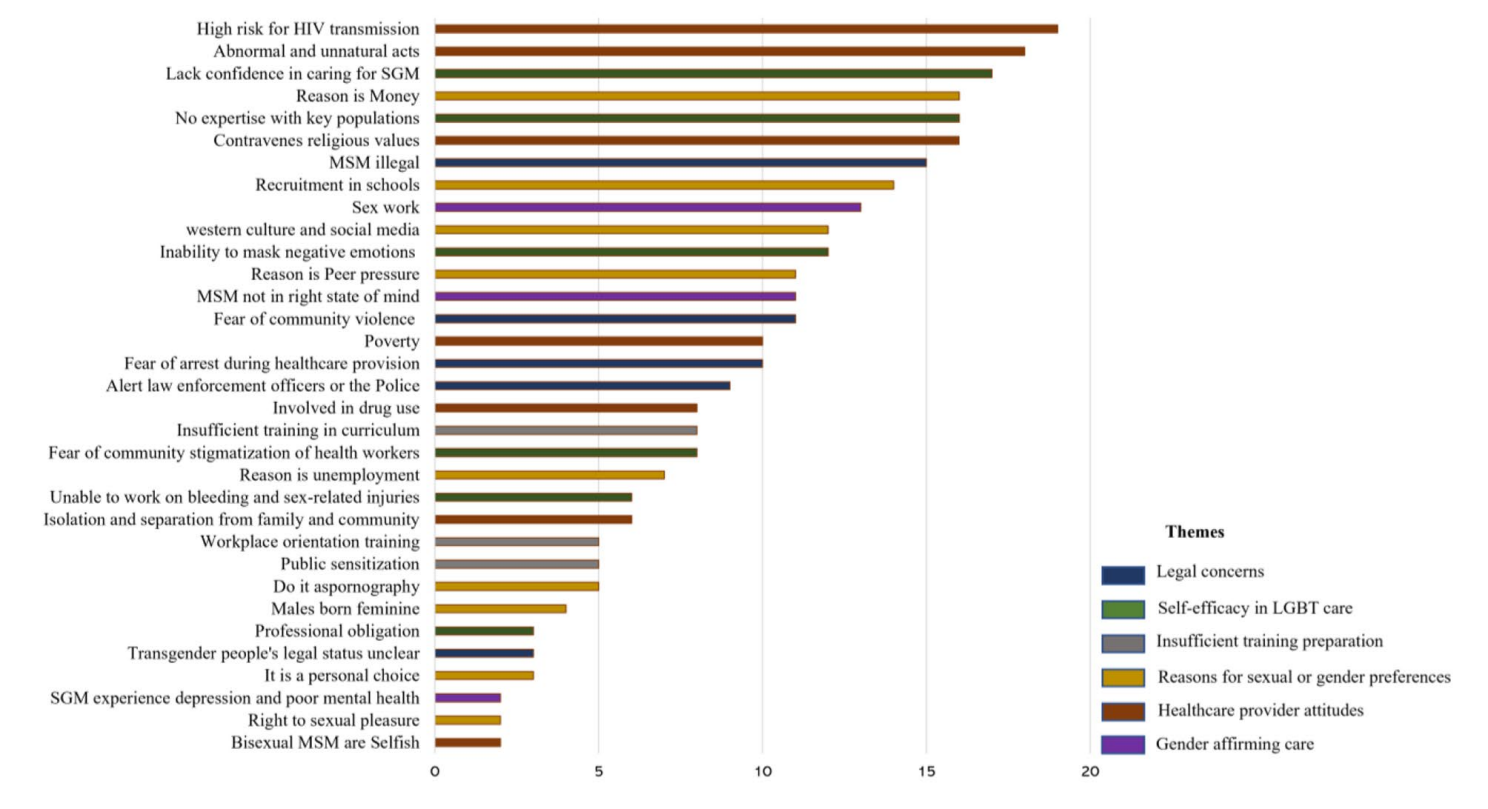
Nurses’ reflections on caring for trans-men after sensitivity training

Eight themes emerged from the analysis: the definition of MSM and TGP, legal concerns, mental illness, healthcare provider attitudes, personal perceptions, self-efficacy in LGBT care, insufficient training preparation, and reasons for gender or sexual orientation preferences (Table 2). These are presented below together with their narrative quotes. The frequencies of key categories and sub-categories are presented in Fig. 1 below and color coded by major category.

**Table 2:** Nurses’ reflections regarding MSM and TGP before sensitization training

Categories	Men who have sex with men (MSM)	Transgender people (TGP)	
**Definition of MSM and transgender**	Gay Homosexual People who have anal sex	**Transgender women** Men who act like women Crossdressers Homosexual	**Transgender men** Male lesbians Homosexual
**Reason for being MSM or transgender**	Personal choice Money Recruitment in schools Peer pressure Western culture Right to sexual pleasure Unemployment	Males born feminine	
**Legal concerns**	MSM Illegal Fear of arrest during healthcare provision Alert law enforcement/Police Fear of community violence		
**Mental illness**	Drug use Depression Not in the right state of mind Sex work		
**Self-efficacy in LGBT healthcare**	Professional obligation No expertise Lack confidence Inability to Mask emotions Bleeding and sex-related injuries Stigmatization of health workers		
**Personal perceptions**	Contravenes religious values Abnormal/ unnatural acts High risk for HIV Isolation and separation Selfishness		
**Attitude in health care provision**	Shock Stigma-Homophobia Unintended disclosure Fear of bodily harm	Confusion Stigma-transphobia Seek advice from colleagues Care in transgender-sensitive health facilities - (non-binary wards, washrooms, and safe spaces) Unwilling to provide care	
**Insufficient training preparation**	Nursing training curriculum Workplace orientation training Public sensitization		

### Definitions

#### Men who have sex with men (MSM)

This was further sub-categorized into being gay or homosexual, anal sex and pain and behaving in a feminine manner.

*Being gay or homosexual.* Most participants (17/19) defined MSM as men who were gay homosexual. They felt that it was a new terminology used in health facilities to diplomatically refer to these populations. They suggested that it was more acceptable for people to say MSM rather than gay or homosexual as mentioned by one participant;

“*MSM is a new word that came in healthcare because generally, we have been regarding them as homosexuals or gay people that’s the general term. a man, having sex with another man instead of a woman*”. (P08, 8 years of clinical practice)

*Having anal sex and pain*. Several nurses reported that whenever someone talks about MSM all they think about is anal sex and pain and they expect that these are some of the services they would be required to provide to these populations.

“*One time I was in church, and the reverend came to preach about homosexuality and [on] a slide show, they had wounds and seemed painful. So, when I think about MSM, I think about anal sex, pain and blood and wounds. I imagine that if they come to the hospital, these are the types of wounds I would have to look at and I am not ready.”* (P05, 2 years of clinical practice)

*Behaving in a feminine manner*. Many nurses defined MSM as men who were feminine. They suggested that usually people who behave in that feminine manner are gay.

*“From the TV, all the homosexuals I have seen behave like ladies. I think they want to be women, that is why they have such type of sex.”* (P04, 2 years of clinical practice)

#### Trans-men

##### Male lesbians

The nurses described them as male lesbians or the masculine partners of lesbians.

*“Transgender man is a male lesbian, those women who have sex with women, but they are the man*”. (P07, 8 years of clinical practice)

*Born female but masculine.* Though the participants could not accurately define who trans-men were, they were able to correctly identify them as assigned female gender at birth.

“*Transgender man is someone who was born female, and usually masculine women, but they now want to be seen and accepted as male*”. (P06, 1 year of clinical practice)

#### Transgender women

##### Cross-dressers

Many participants had not heard of transgender women before. Those who had described transwomen as cross-dressers, people with overstated feminine clothing, men who act as women and as the opposite of being a transman.

“*Crossdressers, with a lot of makeup, big hair wigs, people who are men but act like women, feminine*”. (P08, 8 years of clinical practice)

##### Men in transition

Participants felt that they were not comfortable in their skin thus the use of hormones and surgeries. The nurses suggested that transwomen are hidden or invisible in the community for their safety.

“*They change their body parts, or they do something to make them look like women. [I] heard about hormonal changes and surgeries where body parts are removed and built.*” (P19, 11 years of clinical practice)

#### Reasons for being MSM or trans

The participants had several perceptions regarding the choice to identify as either MSM or transgender. The categories included money, peer pressure, personal choice, recruitment from schools, right to sexual pleasure, unemployment due to COVID-19 and the effects of different cultures and social media.

“*We have been told that there is a lot of money in MSM, so maybe it’s enticing because of the money*.” (P04, 2 years of clinical practice) “*People are doing it as sex work. The jobs are reducing especially now, during COVID many people became unemployed, so you do what you can do to get money*.” (P08, 8 years of clinical practice)

“*Culturally in Africa and Uganda, such things are alien. They learn from the internet especially pornography and videos from social media, and then they try and get addicted and find enjoyment in their new life.”* (P01, 10 years of clinical practice)

For trans-women, specifically, participants felt it was because they were born with a more feminine appearance.

“*Probably I would understand about the trans women, they are born very feminine and probably been bullied and suffered a lot, especially in boys-only schools. Therefore, they just do what is expected and become transgender*.” (P04, 2 years of clinical practice)

#### Legal concerns

One major issue that repeatedly emerged was the legal jeopardy of caring for MSM and TGP.

*“These acts are illegal, we have seen them, they are written about in papers, there is even a bill and even according to the constitution they are illegal.”* (P09, 6 years of clinical practice)

Many nurses suggested they would inform the police and law enforcement for their security because anal sex is illegal in Uganda because of colonial-era laws.

*“Call the police and inform them so that in case of anything, I am protected because at the end of the day if they come to arrest people, they can take me along because I might be seen as an accomplice.”* (P12, 22 years of clinical practice)

Several nurses were afraid of facing arrest while caring for these clients, and fear community violence if people knew they were taking care of MSM and TGP.

*“In Uganda, the anti-homosexuality bill has been going around so I would wonder if I were safe while providing care to this person because anything can happen and before you give your side of the story, you are already in jail.”* (P03, 16 years of clinical practice)

#### Mental illness

Nurses felt that MSM and TGP have mental ill health which caused them to lead that lifestyle or resulted from living that lifestyle.

“*People like homosexuals are mentally ill, they are not in their right state of the mind. This means getting closer to them in a hospital might put me at risk of being raped or beaten up.”* (P15, 5 years of clinical practice)

Many participants suggested that they were not in the right state of mind, which led them to sex work or depression.

*“Sometimes life pushes you to the edge and the only option is sex work. Can you imagine the mental state you have to be in to do gay sex work? I think this leads to a lot of psychological conditions.”* (P02, 5 years of clinical practice)

Others suggested that being MSM or transgender, led to depression, and alcohol and drug use or abuse.

*“These people abuse drugs and alcohol to deal with the things they must face daily. Additionally, the drugs help them to seem dangerous in case they are attacked.”* (P04, 2 years of clinical practice)

#### Personal perceptions

Nurses also expressed their perceptions of MSM and TGP. They felt that these were abnormal and unnatural acts that contravened their religious values and placed these populations at high risk for HIV.

*“If someone came asking me for things that are going to encourage them to do this homosexuality/ MSM like lubricants and condoms, I would give condoms since this is a high-risk group for HIV. I would not give lubricants because that is encouraging the behaviour which goes against my beliefs.”* (P17, 10 years of clinical practice)

Additionally, they felt that some MSM were selfish for having both female and male partners.

*“MSM are causing problems in society because some of them have families, children, and wives yet they have secret male partners as well who are HIV high-risk transmitters. Selfish people, they want to have both women and men, that is not fair.”* (P19, 11 years of clinical practice)

They also suggested that both groups should be isolated and treated separately from other patients to reduce stigma.

*“These people are better off isolated and separated in their hospital because our public facilities are already overwhelmed, and we don’t have time to attend to people who might need extra care and attention.”* (P02, 5 years of clinical practice)

#### Attitude in healthcare provision

The nurses reflected on their attitudes to providing healthcare to MSM or TGP.

##### MSM

Regarding MSM, some nurses feared for their safety and hence were not willing to provide care by themselves.

*“Some of these people are on drugs and other dangerous substances; you don’t want to be stuck alone in a room with such a person. I fear bodily harm like rape or being beaten up. Therefore, I either enter work with a colleague or the client is seen by someone else.”* (P03, 16 years of clinical practice)

Others had homophobic attitudes and imagined they would be shocked if they received such a client.

*“Extremely shocking and unfortunately I would spend more time asking about the individual rather than offering care”.* (P13, 3 years of clinical practice)

Several others were worried about disclosing their status to other family members, work colleagues or even on social media.

*“I don’t think I can work on MSM especially if I know that he has a wife. I am afraid that I would feel obliged to disclose to the family members to warn them and the female partners. These people are at risk of HIV. In this era of social media, all you need to do is post a picture of this gay man and people who know him will inform the family. This is unethical I know, therefore, to prevent this, let them not even come close to me.”* (P04, 2 years clinical practice)

##### Transgender persons

Nurse attitudes towards the provision of healthcare to TGP were mainly those of confusion and seeking advice from colleagues for their safety and security.

*“What comes to my mind is confusion. For example, if I have a trans woman patient, do I place them in the male or female ward? If I send them out for a urine sample, where do I send them, the male or female washroom?”* (P11, 15 years of clinical practice)

Several nurses reported transphobia, recommendation of transgender-specific facilities and an unwillingness to provide care.

*“MSM still looks like a normal man, it’s just their bad sexual behaviours but the trans, I would refer to the national mental referral hospital. I hope that they can offer some trans-specific health care since many of their issues are in mental health.”* (P12, 22 years of clinical practice)

*“I am not willing to offer care to a trans person. I would not even know where to start.”* (P09, 6 years of clinical practice)

### Self-efficacy in lesbian gay bisexual and transgender (LGBT) care

Many nurses reported a lack of confidence and no SGM expertise.

*“They would need to explain a lot because if they just come and say I am transgender or MSM, I would not know where to start; I simply have no expertise.”* (P09, 06 years clinical practice)

Some felt that they were not ready to manage bleeding and sex-related injuries. They could not mask their judgmental emotions and were concerned about stigmatization by work colleagues.

*“I don’t think I am ready to see wounds and whatever. It’s one thing to treat such a person for malaria but another if the condition they have is related to bleeding and sex injuries.“* (P13, 03 years of clinical practice)

The majority said that they would offer care as a professional obligation but only to manage pain or emergencies.

*“It is a professional obligation to give help/care to everyone, so I would want to get them out of the painful situation and that is all.”* (P17, 10 years of clinical practice)

### Insufficient training preparation

One of the nurses’ concerns was that their training doesn’t prepare them to provide healthcare to SGM populations. They felt that some of their perceptions arose from a lack of knowledge about these populations and therefore base many of their perceptions on personal opinions or speculation.

“*During our nursing training, we were never taught about these things. such information should be incorporated in the training curriculum to equip health workers well, especially on how to handle them.”* (P09, 6 years clinical practice)

They recommended the inclusion of MSM and transgender health in nurse training school curricula and in orientation to nursing practice in hospitals to prevent shock and confusion and public sensitization.

*“Information about MSM and transgender care should be incorporated in workplace orientation for new nurses, particularly for facilities that receive these populations. It will prevent the nurses from getting shocked and stigmatizing against them.”* (P04, 2 years clinical practice)

### Nurses’ post-training reflections

The key findings will be presented under three major sections, MSM, trans-men and trans-women (Table 3).

**Table 3:** Coding tree for nurses’ reflections after sensitivity training to reduce stigma towards MSM and TGP in healthcare in Uganda

Population	Theme	Category	Subcategory
**MSM**	Stigma reduction	Increased health care access	Sexuality discussions
			Non-judgmental attitudes
			Anti-stigmatizing environment
		Improved mental health	
	Need for tailored health approaches	Trustworthiness of health providers	
		Sensitization of MSM	
		Incorporate care in existing protocols	
		MSM care is is still a challenge	
		Update health facility registers and forms	
	MSM and the law	A better understanding of the relevant laws	
		MSM and right to healthcare	
		Beneficence	
	Sexual practices and sexuality	MSM and Bisexuality	
		Non-disclosure of sexuality	
		HIV Prevention	Individual HIV prevention
			Community HIV prevention
	Corrected misconceptions	Sexual orientation, gender identity and expression	
		Regular people	
		Individual story and journey	
**Trans-men**	Perceptions and new learning about transmen after the training	Gender identity recognition	Non-judgemental nursing care
			Use of appropriate language and terminology
			Transmen are not male lesbians
			Reduced stigma, discrimination, and barriers to care
			Transgender-sensitive environments
		Reproductive health needs	Menstrual hygiene management
			Pregnancy and parenting
		Social needs	Safe spaces
			Community outreaches
		Safety needs	Legal protection from mob justice
			Personal safety and security
**Trans women**	Gender affirming care	Clinical guidelines and treatment protocols	
		Health training curriculum	
		Hormonal therapy	
	Healthcare provision to transwomen	Deeply rooted beliefs	
		National policy level changes	
		Need for understanding by trans women	
		Professional care	
		Potential violence in inpatient care	
	Need for further training	Counselling for managing the unexpected	
		Intermittent sessions	
		Mentorship and support supervision	
		Practical sessions	
		Traumatizing experience	
	New knowledge acquired	Additional care for trans women	
		Peer trainers and shared stories	
		Self-reflection of prejudice	
	Sexual violence	‌Sexual assault during incarceration	
		Violence during transactional sex	

### Post-training MSM

**Fig. 2:**
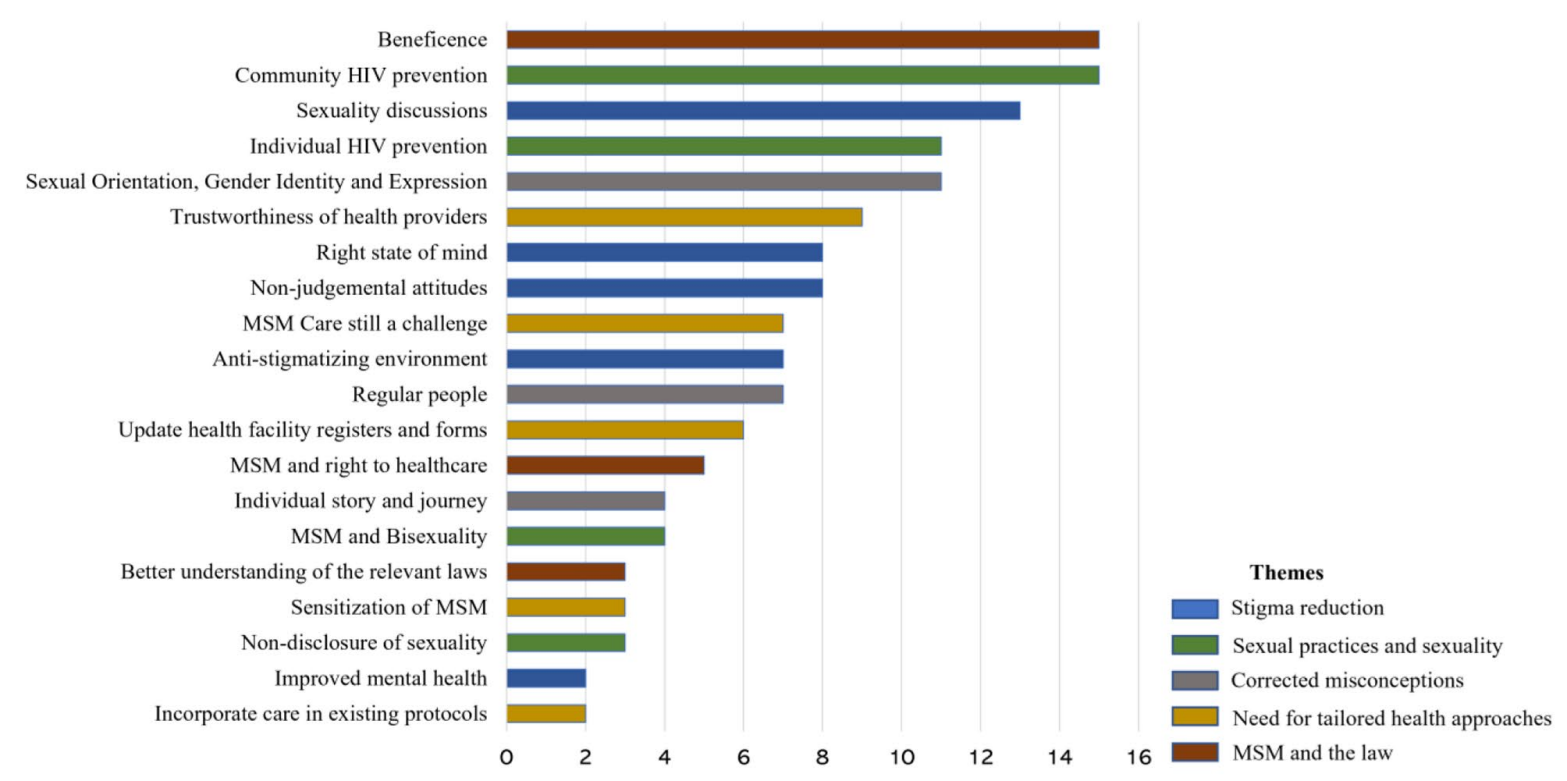
Nurses’ reflections on caring for MSM after sensitivity training

Five themes emerged from the nurse’s reflections on providing care to MSM following the sensitivity training namely ‘stigma reduction’, ‘sexual practices and sexuality, ‘need for tailored health approaches’, ‘MSM and the law’ and ‘corrected misconceptions’. The categories and sub-categories are presented in Fig. 2.

#### MSM and the law

A key reflection that frequently emerged was the understanding that their primary role is to do good and fulfil the pledge that they made while joining the profession. This came through, particularly in HIV prevention.

*“…. the ethical principle of Beneficence and the oath that we took, the pledge of Florence Nightingale, and in Ethics how we have learnt to do no harm and provide care to everybody, so it was a wakeup call.” (P19, 11* years of clinical practice*)*

They also gained a better understanding of the laws that pertain to homosexuality in Uganda.

*“We looked at the Ugandan laws, learning about them helped us understand the relevant laws and the history of some controversial bills. We also realized that we cannot be arrested for offering care*” *(P02, 5* years of clinical practice*)*

Additionally, they reported that MSM are human beings who have a right to access healthcare like any other person.

*“Even though it is illegal, it is not my role to call the police, my role is to offer healthcare. The MSM are Ugandans and in the constitution and other human rights declarations, they have a right to receive healthcare without discrimination.” (P09, 6* years of clinical practice)

#### Sexual practices and sexuality

Nurses expressed a better understanding of MSM sexual practices particularly the bisexuality of many who have families. They expressed concern regarding the non-disclosure of their MSM status to their families which could lead to challenges in HIV prevention. For example, in partner notification programs where a man involves only the female partner. This could hinder both individual and community prevention of HIV.

*“Most are bisexual, so if perhaps one of them is exposed then the other female and male partners in the network and their families are exposed.” (P03, 16* years of clinical practice)

*“MSM look like regular men, so when they come to your facility you will not do the required risk assessment, or they will not disclose which means you will not provide condoms or PrEP [*pre-exposure prophylaxis] *to help with individual HIV prevention.” (P13, 3* years of clinical practice)

#### Corrected misconceptions

The participants acknowledged that before the training they had several misconceptions resulting from a lack of knowledge. The main lesson learned was the difference between sexual orientation and gender identity and expression (SOGIE).

*“I understood the difference between MSM and transgender people because previously, I was putting them in the same category. I thought a transwoman is MSM but from this training, the difference is that MSM is a sexual orientation, but transgender is a gender identity. One can be transgender and heterosexual.” (P11, 15* years of clinical practice)

Additionally, after interacting with, and receiving some sessions from SGM peer trainers, they realized that these are regular people living normal lives.

*“These are regular men; they are working for their living. It means the numbers of MSM reported by the Ministry* [of Health] *may not be true figures, thus, we are not reaching several people who may benefit from key populations targeted programs.” (P5, 2* years of clinical practice).

#### Need for tailored health approaches

The participants highlighted the need for MSM-specific care packages incorporated into mainstream care. Additionally, they suggested that the management of patients could be improved through sensitization and community outreaches.

*“MSM-specific care can be incorporated into the mainstream health services and protocols to get more health workers informed.* The *Ministry of Health should find means of putting this information out there.” (P01, 10* years of clinical practice)

On the other hand, despite the training, some participants still felt incompetent in caring for MSM clients and indicated the need for further training.

*“Dealing with MSM is difficult, based on what we learnt in the training, I still feel that their care remains a challenge even after the training.” (P11, 15* years of clinical practice)

#### Stigma reduction

The main aim of the training was improved sensitivity while working with vulnerable, key and priority populations. Participants acknowledged that they have had stigmatizing attitudes which may discourage MSM from seeking health care. They discussed that stigma could potentially be one of the reasons for depression and other mental disorders that may arise. Therefore, stigma reduction may improve mental health and improve access to health services among MSM.

*“I will not stigmatize them because I now realize that the stigma worsens their mental health state and if they cannot come to the health facility where else will they find refuge?” (P15, 5* years of clinical practice)

They shared several actions nurses may take to reduce stigma including avoiding sexuality discussions, avoiding judgemental attitudes such as preaching anti-gay religious sermons and fostering a welcoming environment.

*“Reducing stigma and making a welcoming environment will help more of the MSM and trans people to come because currently, they fear coming to the health facilities.” (P07, 8* years of clinical practice)

### Post-training: transgender men

**Fig. 3:**
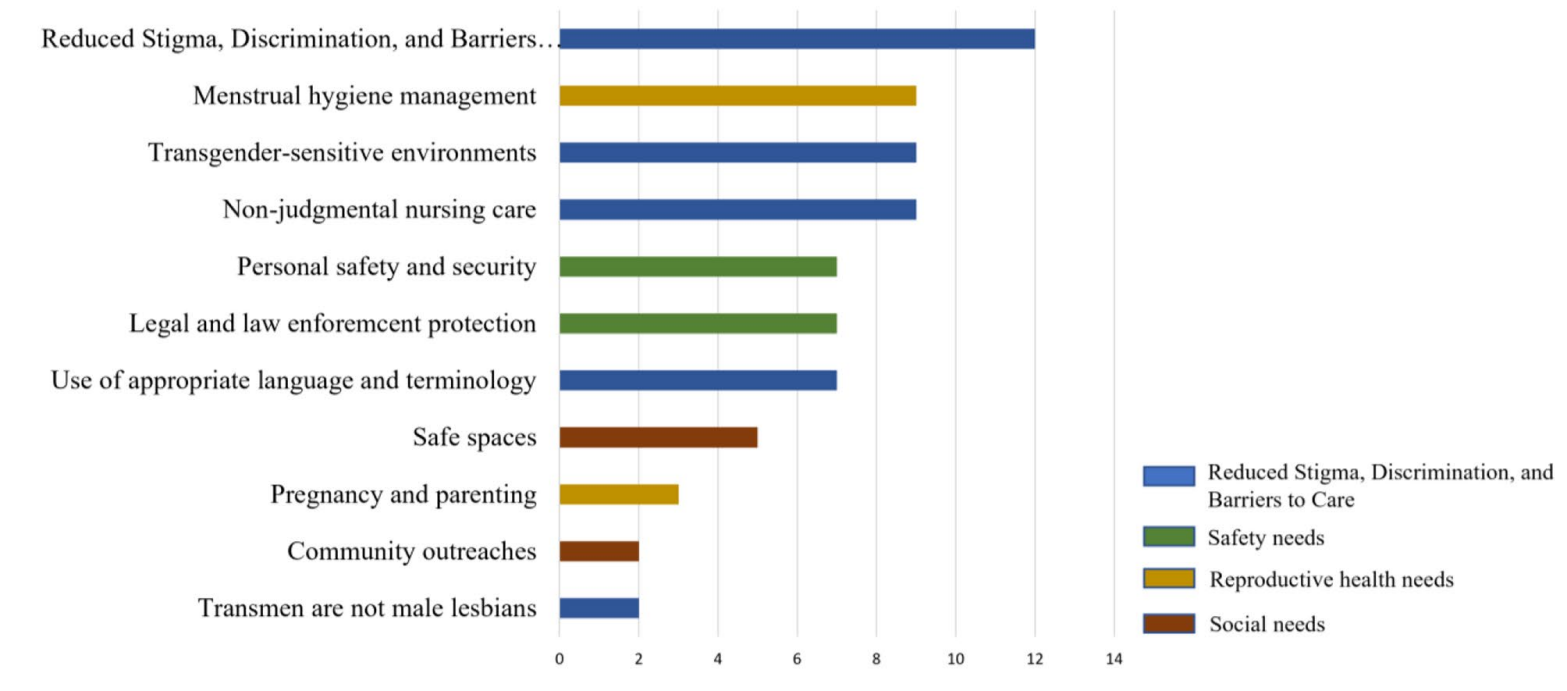
Nurses’ reflections on caring for Trans-men after sensitivity training

Most of the nurses had not heard of trans-men before the training. Therefore, we present their perceptions and new learnings about trans-men after the training. Nurse reflections on caring for trans-men after receiving sensitivity training coalesced around five primary topics: ‘Reproductive health needs’, ‘social needs’, ‘Safety needs’, ‘Gender identity recognition’ and ‘Reduced Stigma, Discrimination, and Barriers to Care’. Figure 3 shows the categories and subcategories and the frequency of mentions.

#### Gender identity recognition

One of the key lessons the nurses learned was the need for gender identity recognition as one of the ways to offer care to both trans-men and trans-women. This included offering non-judgmental nursing care, using trans-appropriate language and terminology such as pronouns and creating transgender-sensitive environments in health facilities.

“*We were advised to endeavour to use appropriate language and terminology and to apologize if we erroneously used the wrong pronoun.”* (P10, 5 years of clinical practice)

The participants also reported an understanding that transmen are not male lesbians as previously thought.

“*We understood the difference between sexual orientation and gender identity and that trans-men are not male lesbians. Those categories are different and classified differently.”* (P19, 11 years of clinical practice)

#### Reproductive health needs

Nurses learned that trans-men who have not yet fully transitioned will continue to experience menstruation and therefore there was a need for support with menstrual hygiene management.

*“One of the unique challenges is the fact that they are before transitioning from female to male, they remain with these female characteristics that involve menstruation. This can be a stressful time for them since they identify as male. There is an unmet need for support with menstrual hygiene management.”* (P11, 15 years of clinical practice)

They also learned that transmen sometimes get pregnant and go on to have children. This further helped in the understanding of SOGIE.

*“The scenarios that they shared around the challenges of pregnancy and parenting because their system is still operating as female. This was a challenge especially if it was an unintentional pregnancy.”* (P01, 10 years of clinical practice)

#### Safety needs

The nurses verbalized an understanding of why TGP are not typically seen in the communities and society because of the potential for assault and injury from members of the public.

“T*here is a need for some assurance of legal and law enforcement protection against mob justice and other forms of brutality.”* (P02, 5 years of clinical practice)

They suggested that the lives of trans-men would be much easier if they were assured of legal protection against mob justice and their safety and security.

*“Male sexual partners may also rape them when they find these people still have their female parts, this may end up sometimes in STIs, HIV or even pregnancy, yet they are identifying as men. Thus, the need for personal safety and security seems paramount.”* (P13, 03 years of clinical practice)

#### Social needs

The nurses noted that trans-men and women will tend to be highly mobile as they search for safe spaces in which to exist in social communities. Therefore, they reflected that community outreach to safe places where TGP can be found for services for example HIV testing, and ART refills may make their care much easier.

“*Conducting community outreaches where you target the bigger population but then you know a smaller population of these people coming through for services might be a better option*.” (P14, 7 years of clinical practice)

### Post-training trans women

**Fig. 4:**
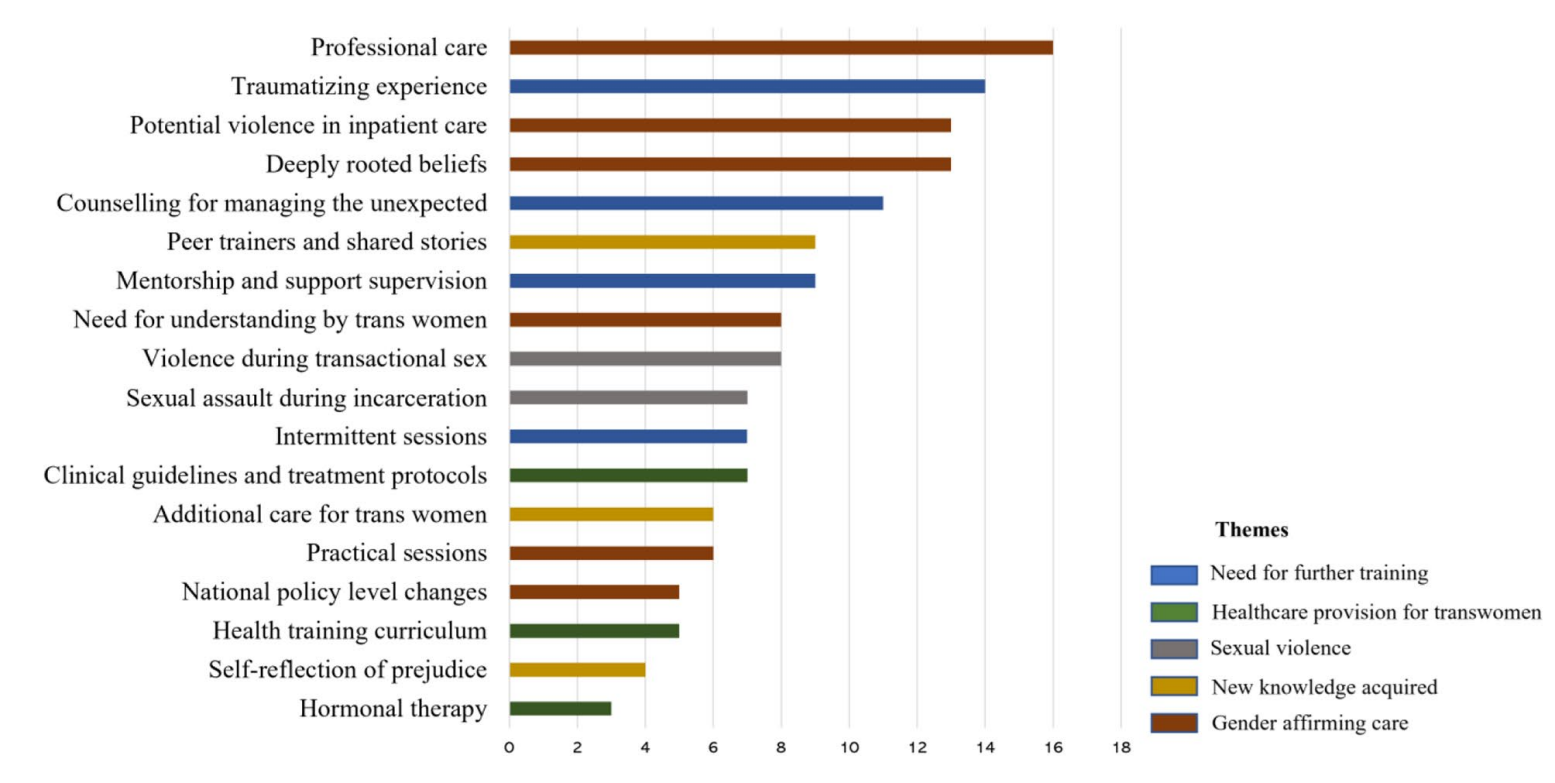
Nurses’ reflections on caring for trans-men after sensitivity training

The nurse’s reflections on caring for trans-women after receiving sensitivity training were around five primary topics: ‘Gender affirming care’, ‘Healthcare provision for transwomen’, ‘Need for further training’, ‘New knowledge acquired’ and ‘Sexual violence’. Figure 4 shows the categories and subcategories and the frequency of mentions.

#### Gender-affirming health care

Gender-affirming health care has been described by Radix, Reisner and Deutsch as “health care that holistically attends to transgender people’s physical, mental, and social health needs and well-being while respectfully affirming their gender identity” [33]. The nurses reflected on the lack of clinical guidelines and treatment protocols or knowledge of specific care for trans-women including hormonal therapy.

*“I was surprised by the high cost of transitioning and hormones. Honestly, if someone even asked me about how these hormones are used, I would not know what to say because we do not have this anywhere in our clinical guidelines or treatment protocols.”* (P10, 5 years of clinical practice)

They also pointed out the gaps in their health training, which could have helped to prepare them to offer this type of care.

*“Hormonal therapy and other gender-affirming care are not part of our health training curriculum. I am not saying that it should be included. I am only saying that I had never heard about it.”* (P16, 15 years of clinical practice)

#### Healthcare provision to trans-women

Nurses reflected that to adequately provide care for trans-women, there is a need for changes in policy at the national level. Additionally, trans-women also need to appreciate that health workers sometimes act the way they do because they lack knowledge of their specialized care.

*“Some of these issues require national policy level changes. For example, if you make transgender sensitive changes and it is a government facility it is violating the government structure.”* (P06, 1 year of clinical practice)

The participants were also aware that they have deeply rooted beliefs that may not change with one training course but appreciated the need to provide professional care.

*“The same way they are saying they can’t change their identity, it’s also difficult for me to remove deeply rooted beliefs so what I can do is to offer professional care at all times”* (P04, 2 years of clinical practice)

The nurses also understood the potential for violence toward trans-women in inpatient care because of the difficulty of whether to place them with males or females.

*“These clients also need our protection. I can imagine if people in the female ward hear the transwoman’s voice it can raise issues and even lawsuits. when you put them on the male side, they may get violated physically or emotionally.”* (P08, 8 years of clinical practice)

#### Need for further training

Some participants reported that the training was a traumatizing experience for them. The nurses felt that there was a need for further training, and they recommended longitudinal intermittent training, especially for nurses who were hearing about these populations for the first time.

*“I am Christian and nurtured to get attracted to the opposite sex, of course, the training helps you a bit, but it was a traumatizing experience. It went against all my beliefs”* (P05, 2 years of clinical practice)

*“It might be better to have intermittent sessions instead of putting everything one go. I felt overwhelmed on most days, and we moved very fast, I almost did not have time to recover.”* (P10, 5 years of clinical practice

They highlighted the need for mentorship and support supervision and counselling for health workers dealing with such unexpected clients. They requested practical sessions where they could meet trans-women in real-world situations in health facilities.

*“Your values and beliefs cannot change so I think the government needs to come up with training sessions to help health caregivers, especially those that are new in serving the key populations. Mentoring them, ongoing supervision and providing psychosocial support.”* (P01, 10 years of clinical practice)

#### New knowledge acquired

One of the key outcomes of the training for the nurses was the new knowledge they gained about offering care to trans-women. Several participants verbalized that trans-women have additional needs that are different from those of either female or male patients. For example, some nurses had not heard of the proctology specialty in Uganda before.

*“Transwomen require additional specialized care. It was my first time to know that there is a specialty in proctology, I didn’t know, and people have even gone to school for it.”* (P16, 15 years of clinical practice)

The shared stories from the peer trainers were eye-opening because nurses were able to ask questions and receive honest answers.

*“I learned more from the peer trainers and shared stories because I was able to ask all the questions that I have always wondered about. They helped us to get the real picture of how they live, what challenges they face and how we can help.”* (P19, 11 years of clinical practice)

Finally, this training was an opportunity for self-reflection on previously unknown prejudices and biases.

*“I realized that I have biases and misconceptions about these people, and I never knew that before. I thought I was an open person. This self-reflection showed me that sometimes our unconscious prejudice can be felt by our patients.”* (P15, 5 years of clinical practice)

#### Sexual violence

The nurses discussed the potential for sexual assault and violence during the times when they are put in jail and during transactional sex from their male clients. The nurses, therefore, suggested training in counselling after sexual violence.

*“The peer trainers shared stories of some of them being sexually assaulted during incarceration by the straight men for example in police cells and prisons.”* (P07, 8 years of clinical practice)

## Discussion

This study aimed to describe nurse reflections before and after attending sensitivity training aimed at stigma reduction towards MSM and TGP at health facilities in Uganda. Most participants had never had a known health encounter with individuals who identified as either MSM or TGP. Before the training, the nurses’ reflections focused on how these populations are defined, their attitudes and self-efficacy in caring for MSM and TGP and legal concerns. There was a positive change in the participant rhetoric after the training workshops. Overall, the nurse’s narrative strongly suggests that several high-level or structural factors determine how they relate to KP. This suggestion is mainly because of the concerns regarding the legal status of MSM and TGP in Uganda. Another critical reflection for the nurses was a reminder of their professional obligation to offer care equitably without discrimination based on sexual preferences and to do no harm to their patients.

Before training, nurses needed to gain relevant knowledge, skills, and training to support MSM and TGP clients. This knowledge gap is likely attributable to a need for more exposure and training in SGM health care. The health professionals’ education does not offer this training which makes healthcare workers inadequately prepared to handle MSM, and TGP needs when they present to their clinics. Our findings agree with prior work in the United States, which found that nurses had limited knowledge about TGP and their specific healthcare needs [34]. Health workers in Kenya reported similar findings before a sensitization training where they reported a lack of professional training with little or no education about MSM health [35]. Similarly, a lack of education on LGBT health issues among nurses and other healthcare providers was reported in several countries [13, 36, 37]. This gap suggests a need to increase knowledge of SGM-specific care in general healthcare settings to mitigate negative experiences [38].

In this study, the nurses reported increased knowledge and an appreciation of the lived experiences of MSM and TGP as they interfaced with health workers after the training. They also better understood the difference between sexual orientation and gender identity and expression after hearing the personal stories of peer SGM trainers. In Kenya, 49% of health workers had adequate knowledge to handle MSM a few months after the sensitization training compared to their baseline scores [18]. In South Africa, there was a significant improvement in the overall knowledge scores of health workers after training which is similar to our findings [20, 39]. Because of the knowledge gained, participants reported increased confidence in dealing with MSM and TGP despite the short length of the training. It remains to be seen whether the confidence level is sustainable. Evidence suggests that insufficient education in medical and nursing schools translates into a lack of knowledge on the job, impacting health workers’ ability to provide medically competent and sensitive care to transgender patients [40]. Therefore, the participants in our study suggested a multi-pronged approach comprising SGM healthcare modules in nurses’ training curricula, intermittent longitudinal sessions with mentorship and support supervision and more practical or real-world scenarios during the in-service sensitivity training. Medical education to address SGM disparities is lacking at universities and other tertiary institutions. Therefore engaging the transgender community members may help in informing medical curricula [41]. On the other hand, some participants in this study reported no change in their attitude towards MSM and TGP despite the training. They reported that they would only offer care as a professional obligation but could not change their deeply rooted spiritual beliefs.

Most nurses reported negative attitudes towards MSM and TGP before the training. Several cited their religious beliefs and culture as the basis for their attitudes. African culture views MSM and TGP as foreign, resulting from external cultures’ influence. Nurses valued their religious beliefs, which viewed LGBT populations as sinners [42]. This perception agrees with our findings that homophobic attitudes are attributable to religious teachings in Uganda, where more than 82% of the population is Christian [43]. A study in Ireland also suggests that many issues of poor health care provision result from the belief that heterosexuality is the preferred mode of sexual orientation [13] among health workers, similar to Uganda’s culture.

Similarly, in Turkey, an assessment of the homophobia scale among nurse educators indicated high levels of homophobia and one of the reasons why nurses do not receive LGBT education during their training [44]. In Zambia, findings from a study among health workers indicated the widespread presence of judgmental attitudes toward LGBT among all cadres of health workers [16]. In the Uganda Penal Code, ‘unnatural offences’ and ‘indecent practices’ are considered offences and punishable by law [45]. Therefore, the nurses were concerned that they might face similar legal action if they knowingly offered care to SGM. However, after the training, nurses reported a better understanding of the relevant Ugandan laws and their role in providing health care to all patients.

Our study reports an overall positive change in the attitude of most nurses and a better understanding of MSM and TGP care after training. This change may have resulted from the information received during the training, which positively affected their attitudes. This assumption agrees with a study in South Africa where health workers were able to provide non-discriminatory and non-judgmental care to MSM after training and believed that sensitization training was an effective intervention to improve awareness of issues regarding TGP and MSM [39]. In another study, homophobic attitudes also decreased three months post-training among health workers in Kenya, particularly those with high baseline scores [18]. This attitude change is also concordant with findings from Canada, which reported that their training intervention showed promise in improving gender-affirming provider knowledge, perceived competency, and attitudes/biases, particularly among those with less transgender and HIV experience [27]. Therefore, researchers and programs in similar contexts may consider training interventions for health workers as a potential SGM stigma-mitigation strategy among health workers. We also recommend an evaluation of the effectiveness and implementation outcomes of these interventions.

### Study strengths and limitations

A strength of our study is the pre-and post-training assessment of nurse attitudes regarding healthcare provision to both MSM and TGP before and after sensitivity training in Uganda. Our study adds to the knowledge base of provider stigma and discrimination in healthcare settings in sub-Saharan Africa. A limitation of the study is that post-training data were collected immediately after the intervention, which did not reflect the long-term effect of training. Additionally, these are the nurses’ reflections before and after the training, not a formal evaluation of the effects of sensitivity training. We acknowledge the authors’ location outside these identities as a limitation in presenting these issues. In that regard, we employed a continuous self-reflection and reflexive approach to aid the researchers in identifying and articulating our standpoint [46, 47]. We met every day and reflected on our positionality and how this potentially influenced the execution of the research.

## Conclusion

Our findings indicate that sensitivity training may help reduce provider stigma and discrimination in healthcare settings and improve service delivery to MSM and TGP. Nursing schools should consider incorporating SGM health training into their curricula to increase awareness of SGM health needs and decrease negative attitudes. There is a need to identify best practices and conduct implementation research to provide culturally sensitive and affirming healthcare delivery in sub-Saharan Africa. Future studies should evaluate the effect of provider sensitivity training on sexual health and HIV outcomes for SGM. Furthermore, interventions targeting higher-level stigma, such as structural and policy levels, are critical because they influence interpersonal stigma reduction efforts and initiatives.

## Electronic supplementary material

Below is the link to the electronic supplementary material.

Supplementary Material 1

Supplementary Material 2

## Data Availability

The datasets used and/or analyzed during the current study are available from the corresponding author upon reasonable request.

## List of abbreviations

KP: Key populations
LGBT: Lesbian gay bisexual transgender
MSM: Men who have sex with men
SGM: Sexual and gender minorities
TGP: Transgender people
