# Supplementary material for: Nurses’ reflections on caring for sexual and gender minorities pre-post stigma reduction training in Uganda
Source: BMC Nurs. 2023 Feb 23;22:50. doi: 10.1186/s12912-023-01208-w (PMC9947888; doi:10.1186/s12912-023-01208-w)
Supplement: Supplementary file 1 — Supplementary Material 1 [file 12912_2023_1208_MOESM1_ESM.docx]

**Supplementary table 1: Nurses’ reflections regarding MSM and TGP before sensitization training**

| **Categories** | **Men who have sex with men (MSM)** | **Transgender people (TGP)** | |
| --- | --- | --- | --- |
| **Definition of MSM and transgender** | Gay  Homosexual  People who have anal sex | **Transgender women**  Men who act like women  Crossdressers  Men who use female hormones  Homosexual | **Transgender men**  Male lesbians  Homosexual |
| **Reason for being MSM or transgender** | Personal choice  Money  Recruitment in schools  Peer pressure  Western culture  Right to sexual pleasure  Unemployment | Males born feminine | |
| **Legal concerns** | MSM Illegal  Fear of arrest during healthcare provision  Alert law enforcement/Police  Fear of community violence | | |
| **Mental illness** | Drug use  Depression  Not in the right state of mind  Sex work | | |
| **Self-efficacy in LGBT healthcare** | Professional obligation  No expertise  Lack confidence  Inability to Mask emotions  Bleeding and sex-related injuries  Stigmatization of health workers | | |
| **Personal perceptions** | Contravenes religious values  Abnormal/ unnatural acts  High risk for HIV  Isolation and separation  Selfishness | | |
| **Attitude in health care provision** | Shock  Stigma-Homophobia  Unintended disclosure  Fear of bodily harm | Confusion  Stigma-transphobia  Seek advice from colleagues  Care in transgender-sensitive health facilities-(non-binary wards, washrooms, and safe spaces)  Unwilling to provide care | |
| **Insufficient training preparation** | Nursing training curriculum  Workplace orientation training  Public sensitization | | |
