# Supplementary material for: Nurses’ reflections on caring for sexual and gender minorities pre-post stigma reduction training in Uganda
Source: BMC Nurs. 2023 Feb 23;22:50. doi: 10.1186/s12912-023-01208-w (PMC9947888; doi:10.1186/s12912-023-01208-w)
Supplement: Supplementary file 2 — Supplementary Material 2 [file 12912_2023_1208_MOESM2_ESM.docx]

**Supplementary table 2: Coding tree for nurses’ reflections after sensitivity training to reduce stigma towards MSM and TGP in healthcare in Uganda**

| **Population** | **Theme** | **Category** | **Subcategory** |
| --- | --- | --- | --- |
| **MSM** | stigma reduction | Increased health care access | Sexuality discussions |
|  |  |  | Non-judgemental attitudes |
|  |  |  | Anti-stigmatizing environment |
|  |  | Improved mental health |  |
|  | Need for tailored health approaches | Trustworthiness of health providers |  |
|  |  | Sensitization of MSM |  |
|  |  | Incorporate care in existing protocols |  |
|  |  | MSM Care is still a challenge |  |
|  |  | Update health facility registers and forms |  |
|  | MSM and the law | A better understanding of the relevant laws |  |
|  |  | MSM and right to healthcare |  |
|  |  | Beneficence |  |
|  | Sexual practices and sexuality | MSM and Bisexuality |  |
|  |  | Non-disclosure of sexuality |  |
|  |  | HIV Prevention | Individual HIV prevention |
|  |  |  | Community HIV prevention |
|  | Corrected misconceptions | Sexual Orientation, Gender Identity and Expression |  |
|  |  | Regular people |  |
|  |  | Individual story and journey |  |
| **Trans men** | Perceptions and new learning about transmen after the training | Gender identity recognition | Non-judgemental nursing care |
|  |  |  | Use of appropriate language and terminology |
|  |  |  | Transmen are not male lesbians |
|  |  |  | Reduced Stigma, Discrimination, and Barriers to Care |
|  |  |  | Transgender-sensitive environments |
|  |  | Reproductive health needs | Menstrual hygiene management |
|  |  |  | Pregnancy and parenting |
|  |  | Social needs | Safe spaces |
|  |  |  | Community outreaches |
|  |  | Safety needs | Legal protection from mob justice |
|  |  |  | Personal safety and security |
| **Trans women** | Gender affirming care | Clinical guidelines and treatment protocols |  |
|  |  | Health training curriculum |  |
|  |  | Hormonal therapy |  |
|  | Healthcare provision to transwomen | Deeply rooted beliefs |  |
|  |  | National policy level changes |  |
|  |  | Need for understanding by trans women |  |
|  |  | Professional care |  |
|  |  | Potential violence in inpatient care |  |
|  | Need for further training | Counselling for managing the unexpected |  |
|  |  | Intermittent sessions |  |
|  |  | Mentorship and support supervision |  |
|  |  | Practical sessions |  |
|  |  | Traumatizing experience |  |
|  | New knowledge acquired | Additional care for trans women |  |
|  |  | Peer trainers shared stories |  |
|  |  | Self-reflection of prejudice |  |
|  | Sexual violence | ‌Sexual assault during incarceration |  |
|  |  | Violence during transactional sex |  |
